# Improved method for extraction and detection *of Helicobacter pylori* DNA in formalin-fixed paraffin embedded gastric biopsies using laser micro-dissection

**DOI:** 10.1016/j.mex.2014.11.003

**Published:** 2014-11-27

**Authors:** María Fernanda Loayza, Fernando Xavier Villavicencio, Stephanie Carolina Santander, Manuel Baldeón, Lourdes Karina Ponce, Iván Salvador, Nicolás Vivar Díaz

**Affiliations:** a Universidad de las Fuerzas Armadas ESPE, P.O. Box 171-5-231B, Av. General Rumiñahui s/n, Sangolquí, Ecuador; b Hospital Carlos Andrade Marín, P.O. Box 170411, Av. 18 de Septiembre s/n y Ayacucho, Quito, Ecuador; c Centro de Investigación Traslacional, Universidad de las Américas, Calle José Queri. Quito, Ecuador; d NETLAB S.A., Calle "A" (Oe7A) N31-145 y Av. Mariana de Jesús, Quito, Ecuador

**Keywords:** Laser micro-dissection, *H. pylori* detection, FFPE tissue, DNA extraction, Gastric biopsie

## Abstract

To assess the molecular events exerted by *Helicobacter pylori* interacting directly with gastric epithelial cells, an improved procedure for microbial DNA isolation from stained hematoxilin-eosin gastric biopsies was developed based on laser micro-dissection (LM) [1]. Few articles have described the use of LM to select and detect *H. pylori* genome from formalin-fixed paraffin embedded gastric tissue [2]. To improve the yield and quality of DNA isolated from *H. pylori* contacting intestinal epithelial cells, the following conditions were established after modification of the QIAamp DNA Micro kit.

• Use of at least 25 cut sections of 10–20 μm of diameter and 3 μm thick with more than 10 bacteria in each cut.
• Lysis with 30 μL of tissue lysis buffer and 20 μL of proteinase K (PK) with the tube in an upside-down position.
• The use of thin purification columns with 35 μL of elution buffer. The mean of DNA concentration obtained from 25 LM cut sections was 1.94± 0 .16 ng/μL, and it was efficiently amplified with qPCR in a Bio Rad iCycler instrument. The LM can improve the sample selection and DNA extraction for molecular analysis of *H. pylori* associated with human gastric epithelium.

## Graphical abstract

**Figure:**
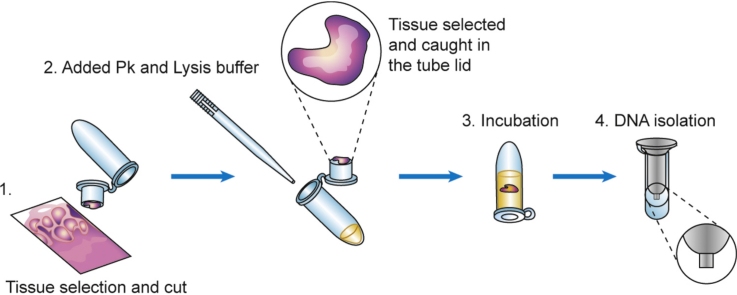

## Method details

Briefly, the improved procedure for microbial DNA isolation from 18 samples included, mounting biopsy tissue in paraffin blocks after fast fixation of gastric biopsy samples. Severity of *Helicobacter pylori* infection was determined after staining paraffin sections with hematoxilin-eosin (HE). Tissue samples with positive diagnosis for *H. pylori* infection were prepared for tissue microarray staining with Wartin–Starry silver stain [2] followed by laser micro-dissection (LM) of region of interest. DNA extraction and amplification was performed on micro-dissected samples.

## Subject selection

Study subjects were selected based on the presence of the following dyspeptic symptoms: postprandial fullness, early satiation, epigastric pain, epigastric burning, nausea or abdominal bloating; which occur in the absence of an organic cause that readily explains them [3]. Subjects with dyspeptic symptoms, who provided informed consent to extract and use tissue biopsies for conventional pathology studies and molecular analysis were included. To obtain a high quality sample from tissue biopsies, tissue had to be immediately fixed in a formalin 10% (pH 7.0) solution for no more than 8 h. Then, the conventional procedure for histo-pathological analysis was applied and samples were mounted in paraffin blocks [1]. *H. pylori* load in tissue samples was determined based on the following semi quantitative criteria [2], (a) negative (no bacteria cells were observed/field = −), (b) mild (0–10 bacteria/field 1000× = **+**), (c) moderate (11–30 bacteria/field 1000× = **++**) and (d) severe (more than 31 bacteria/field 1000× = **+++**). An experienced pathologist with more than 30 years of experience analyzed all biopsy samples. Only samples reported with moderate and severe infection were used for LM.

## Sample preparation

**Fig. 1:**
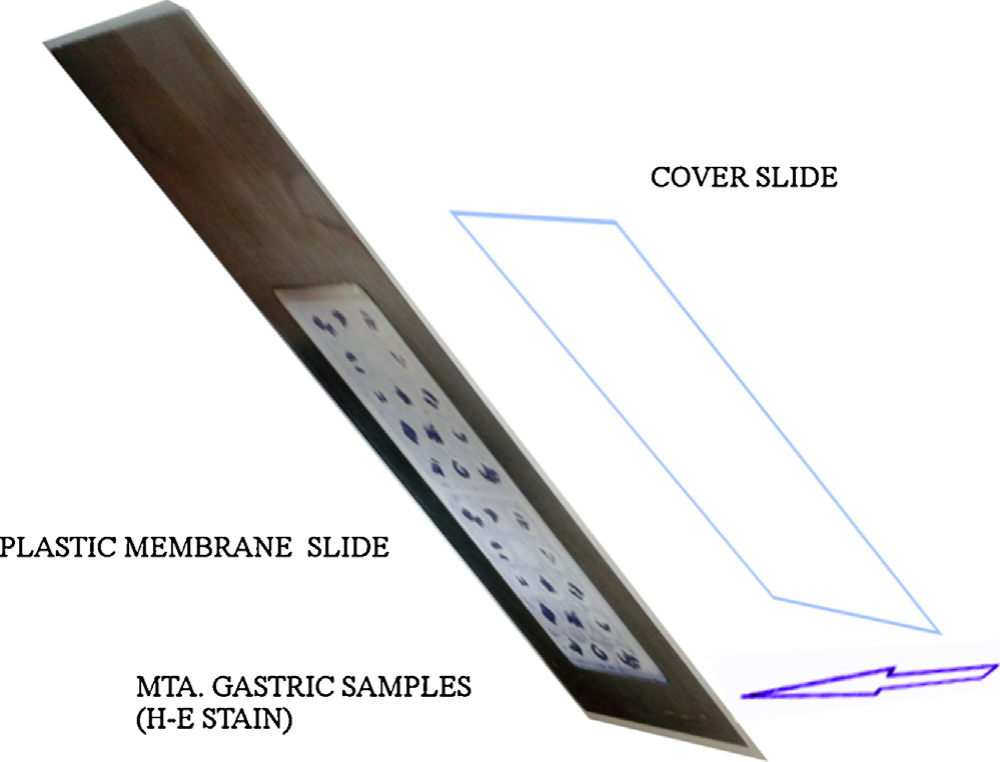
Molecular-grade membrane mounted slide (MMI Cell Cut Plus^®^ System, OLYMPUS; Record Dental, Quito, Ecuador) with two sections of microarray block. This figure explains how the cover slide should be mounted.

**Fig. 2:**
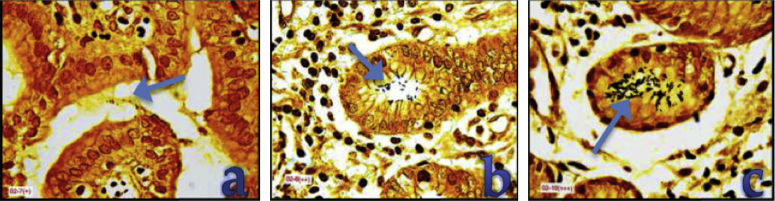
Gastric Biopsy sections with Wartin–Starry silver stain. (a) Mild infection (+) 0–10; (b) moderate (++) 11–30; and (c) severe infection (+++) >31 bacteria/field. The bacteria are indicated by the blue arrow.

**Fig. 3:**
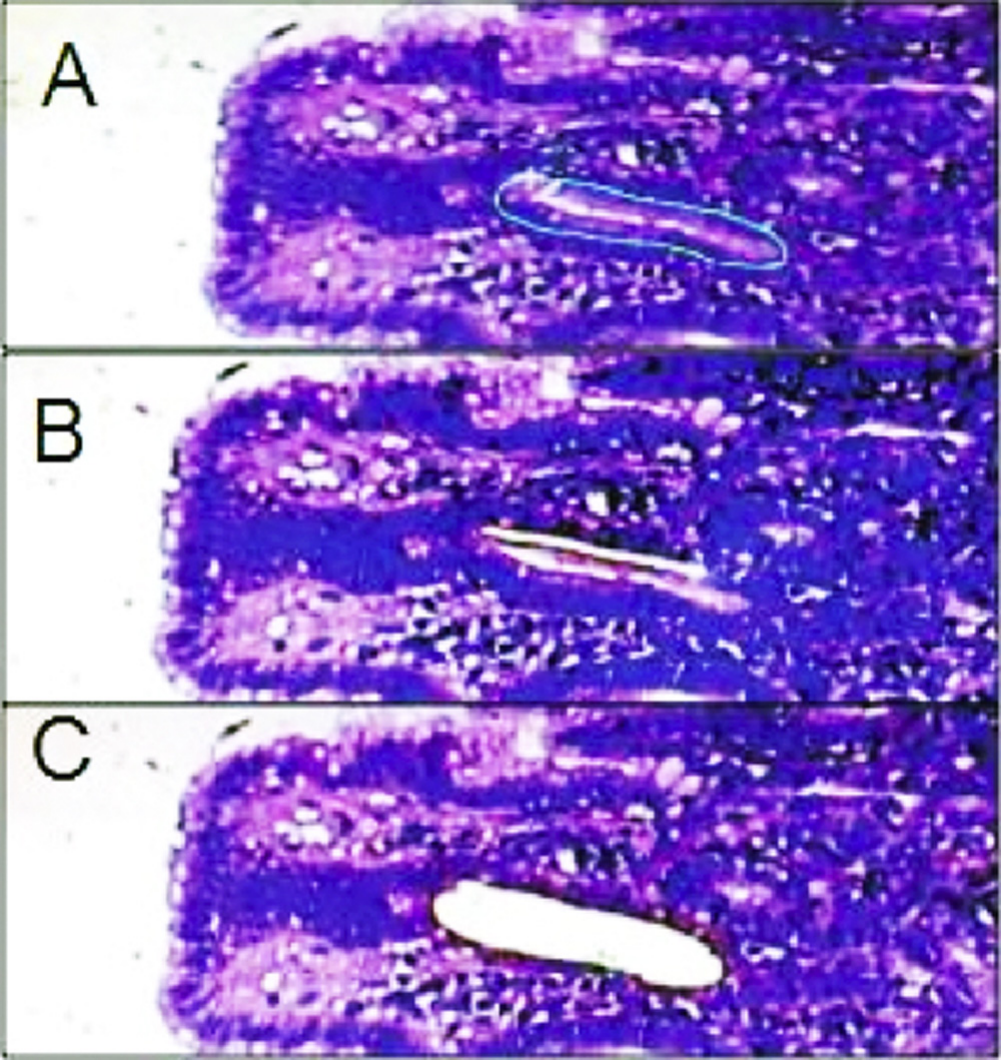
Hematoxilin–Eosine (HE) stained plastic slide used for LM procedure. (A) Software allowed the user to pre-select the cut zones. (B) The laser cut is evident in the tissue on the plastic slide just around the circled figure. (C) The image shows a view of the tissue after the laser cut and is captured by the adhesive lid of a tube.

1. Tissue micro arrays (TMA) were prepared from the formalin-fixed paraffin embedded (FFPE) tissue blocks that were positive for *H. pylori* infection using the QuickRay^®^ Master UATM 272. Three cylinders of 3 mm in diameter were taken from each patient’s block. The cylinders were placed in a recipient block (QuickRay® UNITMA System). In order to obtain negative tissue controls, two FFPE blocks reported negative for *H. pylori* infection were used.
2. Each tissue array block was placed in a paraffin tissue-embedded station (HistoStar™, ThermoScientific). Subsequently, three sections of 3 μm of thickness, from the tissue micro array block, were cut with a new disposable blade in a semiautomatic microtome (Shandon Finesse Me+, Thermo Scientific). The block tape obtained was placed on the surface of a tissue flotation water bath at 45 °C. The tape was gently stretched until the tissue contained on the tape had no wrinkles.
3. Two of the three adjacent block tapes were collected on a molecular-grade membrane mounted plastic slide (mmi Cell Cut Plus^®^ System, OLYMPUS).
4. The plastic slide was placed on a DAKO AutoStainer to perform the HE conventional stain [2]. To facilitate the capture of the cut section by LM, a cover slide was placed in the back of each sample slide (Fig. 1). After the LM cut, a tube with adhesive in the lid (mmi Cell Cut Plus^®^ System OLYMPUS) was used to pickup the sample.
5. The third adjacent block tape was placed on a polilysin-positive charged slide [1] and was processed in an Artisan Link Special Staining System for Wartin–Starry silver stain [2] (Fig. 2). This procedure allowed the use of high-resolution image microscopy (VIRTUAL; OLYMPUS) to identify the bacteria interacting with gastric epithelial cells. Thus, this third section was used to guide the selection of the area for LM in the HE stained samples [2] (Fig. 2).
6. Laser micro-dissection was performed in samples with moderate or severe bacterial load: (more than 10 bacteria/field 1000×). The region to cut was defined by the bacteria load in each optical field analyzed. Variation in the number of bacteria that could be observed in the gastric surface is expected. Fig. 3 shows sample processing for the selection of *H. pylori* associated with gastric epithelial cells from the HE stained samples [2]. The size of the closed drawings were approximately 10–20 μm in diameter. The selected zones targeted the lumen of the gastric gland, always avoiding human cells nuclei [2] (Fig. 3).
7. In order to obtain negative tissue controls, 4 gastric samples reported as “negative” for *H. pylori* infection, using HE and Warthin–Starry silver stain, were chosen. More than 25 cuts with LM were performed in the mucus layer of gastric epithelial cells avoiding the nucleus of human cells.

## DNA extraction

**Fig. 4:**
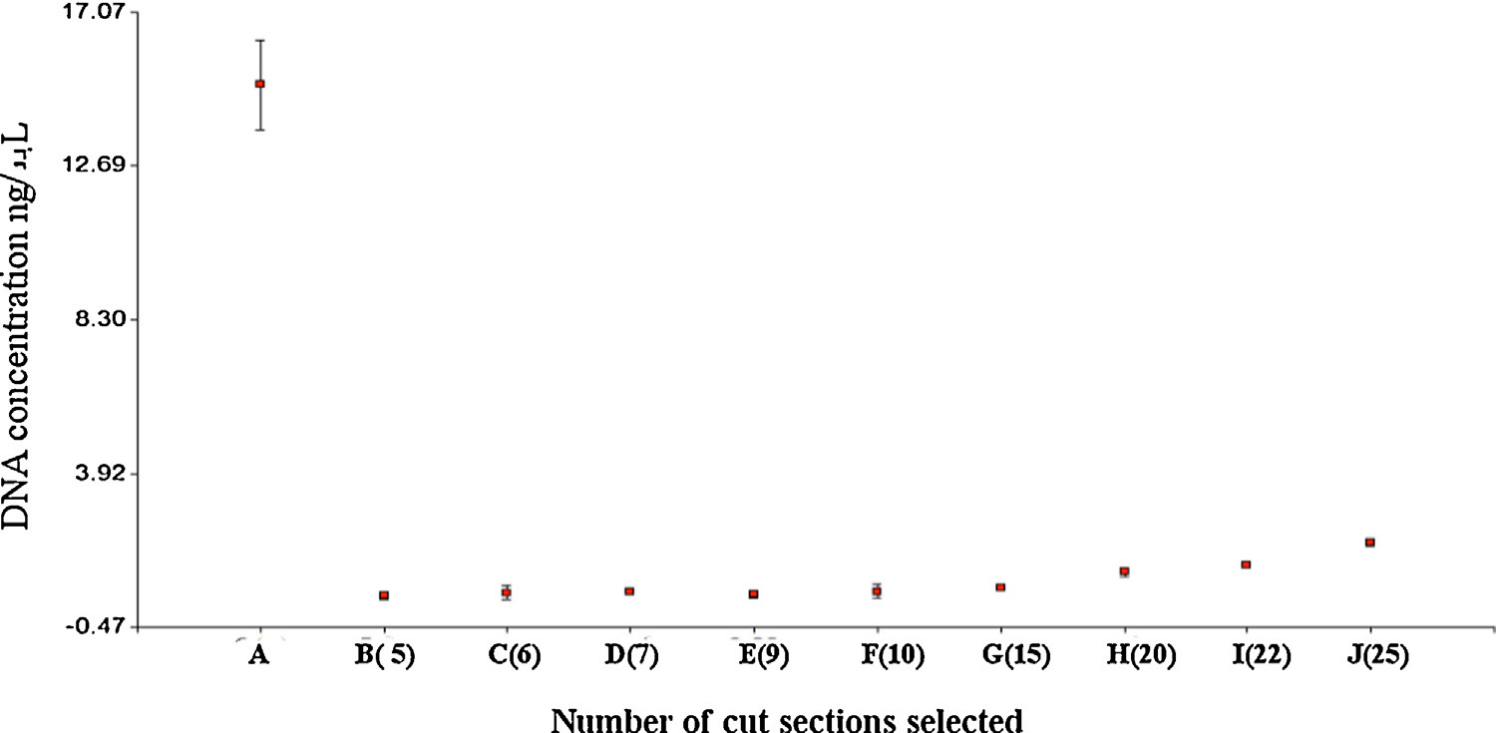
Point A shows the DNA concentration that was isolated from 5 tape cuts from FFPE gastric tissue block (each tape was 3 μm thick). Points B–E along the *X*-axis represent the number of LM cut sections (between 5 and 25), with more than 10 bacteria added to the gastric surface. The cut sections were added to the cap of a 0.2 mL tube and were used to perform DNA isolation assay. The *Y*-axis shows the DNA concentration, reported in ng/μL.

**Fig. 5:**
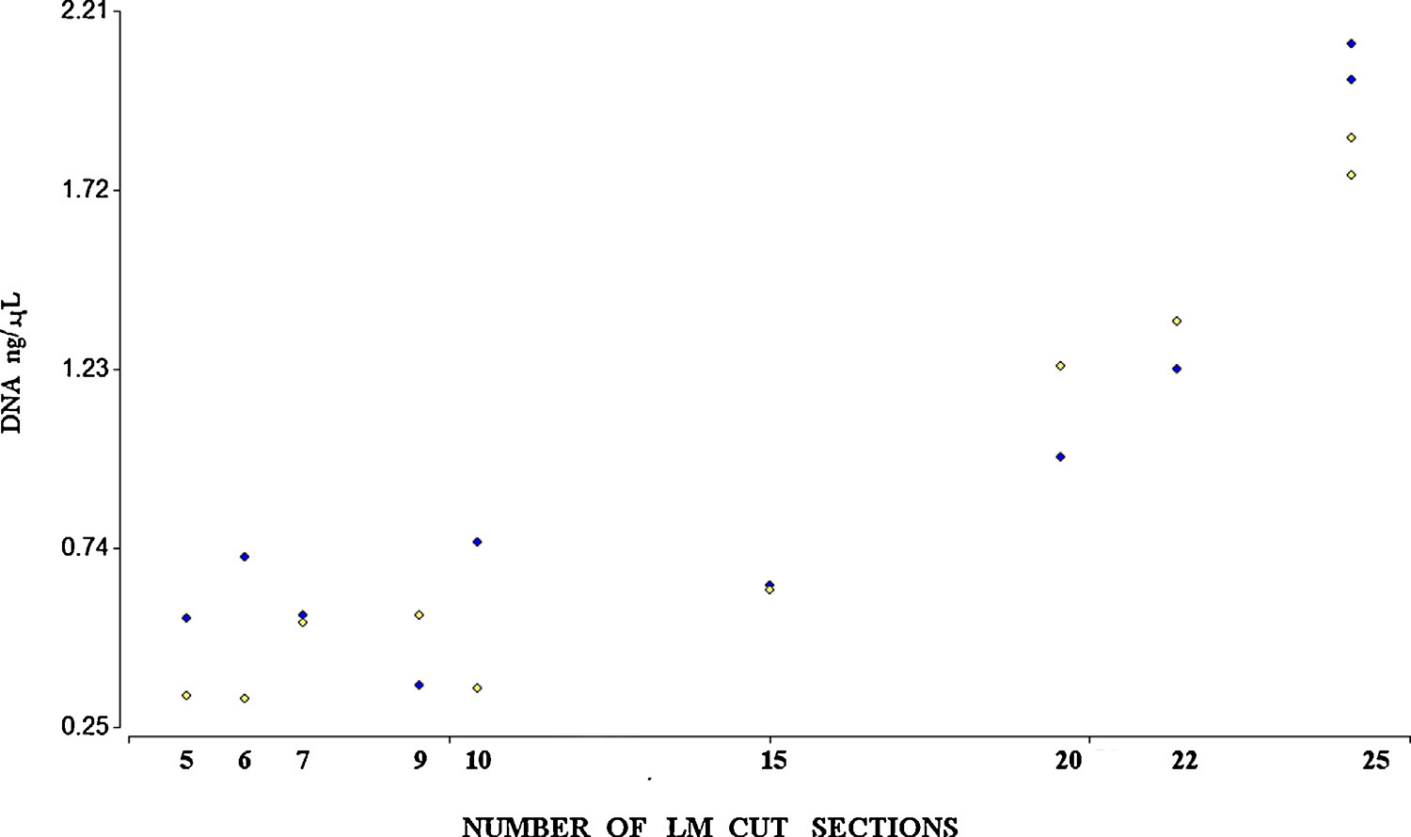
The DNA concentration (ng/μL) obtained from samples with different numbers of LM cut sections. The blue dots represent first (blue) and second (yellow) replication.

In order to determine the minimum amount of samples to obtain sufficient DNA for analysis, we collected 5–25 LM separately cut sections and processed them for *H. pylori*-DNA isolation. The extraction procedure was performed with QIAamp DNA Micro Kit (Quiagen) with the following modifications:

1. 30 μL of tissue lysis buffer (ATL) and 20 μL proteinase K were added to each laser micro-dissected sample collected in a 0.2 mL tube.
2. The samples collected were mixed in a vortex for 15 s and then placed in a thermo-block with the lid down because the sections of tissue were attached to the top (Graphical abstract). Samples were incubated for 6 h at 56 °C with occasional vortexing pulses. Then, 50 μL of ATL were added. Each sample was mixed and the lid reviewed with a magnifying glass to confirm that all sample sections had been lysed.
3. The complete material was transferred to a new 1.5 mL tube. Then 100 μL of working AL solution (2 μL of CAR was mixed with 98 μL Buffer AL) was added and mixed in a vortex for 15 s.
4. Then, 100 μL of molecular-grade ethanol was added and mixed in a vortex for 15 s. The sample was incubated for 5 min at room temperature.
5. The sample was then centrifuged in the spin mode for 10 s to remove drops from inside the lid.
6. Approximately 300 μL of the lysate sample was transferred to the QIAamp MinElute column placed into a 2 mL collection tube. The sample was centrifuged at 8000 rpm for 1 min and the column was placed in a new tube.
7. The column was then washed with 700 μL Buffer AW1 and centrifuged at 8000 rpm for 1 min. The column was once again placed in a new collection tube.
8. Then 700 μL of AW2 buffer was added to the column and the centrifugation protocol was repeated to discard the AW2 buffer.
9. The QIAamp MinElute column, adapted to a new 2 mL collection tube, was centrifuged at 14,000 rpm for 3 min to dry the membrane.
10. The last collection tube was replaced with a 1.5 mL tube (previously labeled with sample identification number).
11. Then 35 μL of the AE Buffer was dispensed in the center of the column. After 5 min incubation at 20–25 °C, the sample was centrifuged at 14,000 rpm for 1 min.
12. The isolated DNA in the 1.5 mL tube was stored at −20 °C until assayed. The DNA was quantified with Picogreen, (INVITROGEN™) following standard instructions recommended by the manufacturer.
13. To isolate DNA from the conventional FFPE, five tape sections were cut with a new disposable blade in a semi-automatic microtome (Shandon Finesse Me+, Thermo Scientific) and were directly collected in a 1.5 mL sterile Eppendorf tube. Then DNA extraction was performed according to suggested manufacturer instructions (QIAamp DNA Micro Kit Quiagen).
14. Data showed that the greatest number of tissue sections obtained with LM yielded greatest concentrations of DNA. The average DNA concentration obtained from 25 LM cut sections was 1.94 ± 0.16 ng/μL. The DNA concentration obtained from LM samples was 10-fold less than the concentration obtained following conventional extraction from FFPE gastric biopsies (Fig. 4). Fig. 5 indicates the consistency of the method to isolate microbial DNA. Although the final concentration of DNA isolated by this method is less than the amount of DNA isolated by conventional strategies, the obtained nucleic acid is of bacterial origin.

When DNA is isolated directly from FFPE tissue blocks, the sensitivity of amplification and detection of *H. pylori* genes could be reduced up to 10 times [4]. It is important to take in consideration that the colonization and gastric infection can be caused by more than one genotype of *H. pylori* [5]. With this method only DNA from bacteria that was associated with epithelial cells was isolated. This improvement could help to study the specific molecules which take part in the pathophysiology of the infection.

## DNA amplification

**Fig. 6:**
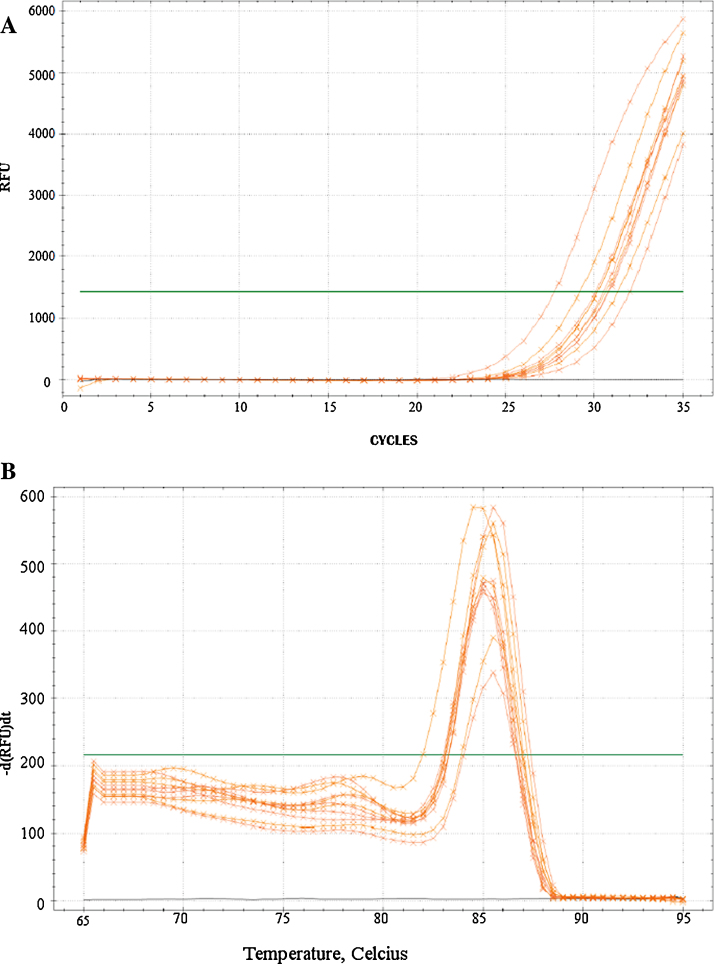
qPCR results for detection of 16S rRNA gene region of *Helicobacter pylori* with HP6 primers in (a) Log Amplification and (b) melting curves.

The suitability and quality of isolated DNA to study *H. pylori*, were evaluated with qPCR amplification [1], [2]. For this, primers for a 222 bp region of *H. pylori* 16S rDNA partial gene were generated. Specifically, forward (FW) and reverse (RV) primers were designed in Blast software to guarantee the specificity of amplification of *H. pylori* 16S rDNA, minimizing the possibility to amplify any fragment of human genome. Three primer pairs were tested using DNA isolated from a cultured *H. pylori* strain, three tissue samples with positive reports of *H. pylori* infection and negative controls (data not shown). The analysis for “primer” selection consisted on: an optimal reaction efficiency and specific melting curves of the qPCR reaction. Also, as *H. pylori* is a pathogen that can survive to the hostile environment of the gastric tissue, there is low probability of contamination with other bacterial pathogens [6]. The primers selected for analyses were HP6 forward (5′-AGACACGGTCCAGACTCCTA-3′) and reverse (5′-ACGCCCAGTGATTCCGAGTA-3′) based on the best melting curve and reaction efficiency (Fig. 6). The amplification program is described in Table 1.

Eighteen samples were amplified with HP6 primers. The results showed two possible 16S rDNA variants based differences in melting curves temperatures. A group of 12 samples exhibited a melting temperature of 85.5 ± 0.5 °C and six samples reported a melting temperature of 83.5 ± 0.5 °C (Table 2).

In conclusion, an efficient DNA extraction process was applied for LM section samples in order to study *H. pylori* directly associated with the gastric epithelium. The study of *H. pylori* in proximity to gastric epithelium could better reflect the pathophysiology of the interaction between the bacterium and the host epithelium than the analysis of *H. pylori* without known association. There are research reports that show the relevance of direct interaction between the type IV secretion system from *H. pylori* and the surface of host cells [7]. This complex structure is required for translocation of CagA cytotoxin into the gastric epithelial cells [7]. Some of the consequences of this interaction have been described, but there are many more questions to answer about the relationship between bacteria structures and cytotoxin translocation with host signaling molecules and the specific host cell response.

The severity of the gastric disease symptoms depends on the genetic diversity of *H. pylori* strain that can colonize the gastric epithelium [8]. An Ecuadorian research study on patients with different severity of gastric disease showed that the intimate interaction between bacteria directly associated to the epithelial host cells was different from the interaction between bacteria that were in the mucus layer (non published data). It is possible that particular genotypes of *H. pylori* show more affinity to the particular host cells than others. The use of LM and specific DNA isolation from *H. pylori* associated to host cells surface will clarify the cellular microbiology of this infection.

## Figures and Tables

**Tabla 1:** qPCR amplification program for 16S rRNA (Hp1 and Hp6) of *Helicobacter pylori*[9].

Phase	*T*°	Time
Initial denaturation	95 °C	10 min
Denaturation	94 °C	30 s
Anneling	51 °C	1 min
Elongation	72 °C	1 min
Final elongation	72 °C	10 min
Cycle number	35	2 h 22 min

**Table 2:** Melting temperature for 16S rDNA amplification with HP6 primers for 18 samples tested.

Result	Melting temperature	*n*
Positive 16S DNA	85.5 ± 0.5 °C	12
Positive 16S DNA	83.5 ± 0.5 °C	6
Negative 16S DNA	NA	4
	Total	22
